# Adaptation of WHO’s generic tuberculosis patient cost instrument for a longitudinal study in Africa

**DOI:** 10.1080/16549716.2020.1865625

**Published:** 2021-01-25

**Authors:** Denise Evans, Craig van Rensburg, Caroline Govathson, Olena Ivanova, Friedrich Rieß, Andrew Siroka, Abdou K. Sillah, Nyanda Elias Ntinginya, Ilesh Jani, Farzana Sathar, Sydney Rosen, Ian Sanne, Andrea Rachow, Knut Lönnroth

**Affiliations:** a Health Economics and Epidemiology Research Office, Department of Internal Medicine, School of Clinical Medicine, Faculty of Health Sciences, University of the Witwatersrand, Johannesburg, South Africa; b Division of Infectious Diseases & Tropical Medicine, Klinikum of the University of Munich, Munich, Germany; c Health Financing Department, World Health Organization, Geneva, Switzerland; d Medical Research Council Unit, The Gambia at the London School of Hygiene and Tropical Medicine, Fajara, The Gambia; e NIMR - Mbeya Medical Research Centre, Mbeya, Tanzania; f Instituto Nacional de Sauúde (INS), Maputo, Mozambique; g Aurum Institute, Johannesburg, South Africa; h Department of Global Health, Boston University School of Public Health, Boston, MA, USA; i Clinical HIV Research Unit, Department of Internal Medicine, School of Clinical Medicine, Faculty of Health Sciences, University of the Witwatersrand, Johannesburg, South Africa; j Department of Global Public Health, Karolinska Institute, Stockholm, Sweden

**Keywords:** Catastrophic total costs, income, pre-treatment, out-of-pocket, TB sequelae

## Abstract

The WHO developed a generic ‘TB patient cost survey’ tool and a standardized approach to assess the direct and indirect costs of TB incurred by patients and their households, estimate the proportion of patients experiencing catastrophic costs, and measure the impact of interventions to reduce patient costs. While the generic tool is a facility-based cross-sectional survey, this standardized approach needs to be adapted for longitudinal studies. A longitudinal approach may overcome some of the limitations of a cross-sectional design and estimate the economic burden of TB more precisely. We describe the process of creating a longitudinal instrument and its application to the TB Sequel study, an ongoing multi-country, multi-center observational cohort study. We adapted the cross-sectional WHO generic TB patient cost survey instrument for the longitudinal study design of TB Sequel and the local context in each study country (South Africa, Mozambique, Tanzania, and The Gambia). The generic instrument was adapted for use at enrollment (start of TB treatment; Day 0) and at 2, 6, 12 and 24 months after enrollment, time points intended to capture costs incurred for diagnosis, during treatment, at the end of treatment, and during long-term follow-up once treatment has been completed. These time points make the adapted version suitable for use in patients with either drug-sensitive or drug-resistant TB. Using the adapted tool provides the opportunity to repeat measures and make comparisons over time, describe changes that extend beyond treatment completion, and link cost survey data to treatment outcomes and post-TB sequelae.

**Trial registration**: ClinicalTrials.gov: NCT032516 August 1196, 2017.

**Abbreviations**: DOTS: Directly observed treatment, short-course; DR-TB: Drug-resistant tuberculosis; MDR-TB: Multi-drug resistant tuberculosis; NTP: National Tuberculosis Programme; TB: Tuberculosis; USD: United States Dollar; WHO: World Health Organization.

## Background

The economic burden on households and individuals of illness due to TB can be devastating. Costs borne by patients can have catastrophic consequences, potentially entrenching individuals and households in a vicious poverty-disease cycle [1,2]. One of the three targets of the WHO End TB Strategy is that no TB patients or their households should face catastrophic total costs due to TB disease [3]. Catastrophic total costs is defined as costs (including direct medical expenditures, non-medical expenditures and overall indirect costs – which includes loss of paid work and/or time off work necessitated by symptoms and treatment seeking) that account for 20% or more of the patient’s annual household income [4,5].

To reduce the risk of TB-related impoverishment, it is therefore important to document the types, magnitude, and drivers of TB-related costs for patients and their households so that appropriate policies and interventions, such as health financing, patient-centered delivery models, and social protection mechanisms (e.g. job protection, paid sick leave, social assistance, cash transfers, etc.) can be developed [2,6]. In 2015, WHO established a standardized protocol for conducting nationally representative TB patient cost surveys that assess the direct and indirect costs incurred by TB patients and their households to determine the proportion of TB patients who experience catastrophic total costs due to TB disease. In 2017, based on field-testing of the generic TB patient cost tool in nine countries and after consultation with a WHO-led TB Patient Cost Task force, the protocol was revised and expanded into a handbook [2]. By March 2020, 17 countries have completed the survey and an additional 30 were planning one in 2020 [7]. Countries that have completed analyses estimate that 27% to 83% of patients with any form of TB experience catastrophic costs. This number is much higher, at 67% to 100%, among those with drug-resistant TB [4].

The WHO instrument was designed for a cross-sectional, national, facility-based survey of patients registered for TB treatment in a country’s National Tuberculosis Programme (NTP) who are attending a sampled facility for a visit during the survey period. Patients are interviewed only once, during either the intensive or the continuation phase of treatment [2]. Data collected in either the intensive or continuation phase are extrapolated to estimate the total costs for the entire TB episode.

While previous surveys have provided valuable information [2], the cross-sectional design has several limitations beyond those that are common to any survey. In particular, costs incurred before treatment are ascertained through recall interview, and only for those patients interviewed in the intensive phase of treatment. Costs incurred after treatment completion are missed, and costs cannot be related to treatment outcomes, which are usually not available at the time of survey implementation. The analysis is complex and involves extrapolations and imputations to calculate the total costs incurred for the entire duration of TB. As a result, several TB patient cost studies have highlighted the need for longitudinal studies [6,8]. There are a few longitudinal studies underway; however, none of these are collecting patient costs after TB treatment completion. This is likely to underestimate the economic burden of TB as costs incurred after the completion of treatment, due to TB sequelae or loss of productivity due to disability, would be ignored [9]. Similarly, economic recovery (e.g. ability to pay back loans or regain productivity) and the ability to build resilience to future shocks after completing TB treatment are essential consequences that should be measured to understand TB’s full economic impact.

TB Sequel (NCT03251196) is a multi-country, multi-center, observational cohort study designed to understand the pathogenesis and risk factors of long-term sequelae of pulmonary TB in South Africa, Mozambique, Tanzania, and The Gambia [10]. The primary outcome is the proportion of TB patients with severe lung impairment measured by spirometry at 24 months after TB treatment initiation. There are four sub-studies nested within the main cohort study, and one of these is the ‘Socio-economic costs and Impact of TB’ sub-study, which aims to determine the occurrence of reversible and irreversible socio-economic consequences of TB on patients and their households before, during, and after TB treatment [10]. For this sub-study, patient interviews are conducted at study enrollment (Day 0 ± 14 days) and 2, 6, 12, and 24 months after enrollment. Further details about data collection and tools are available elsewhere [10]. To overcome some of the limitations of the existing WHO generic TB patient cost survey, we took advantage of TB Sequel to adapt the existing cross-sectional instrument for a longitudinal study design and tailored it for each of TB Sequel’s study countries. Here we describe the process of creating a longitudinal instrument and its application in the study.

## Methods

### Original instrument

The WHO generic TB patient cost survey instrument collects information from patients on current TB treatment and costs incurred during the TB treatment phase the patient is interviewed in [2]. Costs are then calculated for the assumed total treatment duration through extrapolations and imputations (Table 1). Patients in the intensive phase of treatment are asked also about costs incurred before treatment. The survey instrument collects data on the patient’s demographic (e.g. age, gender, employment status, household composition, etc.) and economic position (e.g. individual and household income, household assets, household food security, etc.), direct out-of-pocket medical (e.g. consultation fee, laboratory tests, medication, etc.) and non-medical (e.g. food, accommodation, transport) payments, indirect costs (i.e. income loss or time cost as a result of TB), and guardian costs. Patients’ estimates of expenses for nutritional/food supplements, health insurance reimbursements, social assistance/protection (i.e. welfare, disability grants, cash transfers, etc.) and social consequences of TB are also collected. The patient is also asked about the financial consequences of TB and how they cope with these (i.e. dissaving, borrowing funds, selling assets to cover the costs of health-care expenditure). This instrument is designed to be administered only once per patient over the course of treatment. Before the interview, patient information is obtained from the TB treatment card and participants provide informed consent.

**Table 1.** Characteristics and limitation of the WHO TB patient cost survey and the adapted version for longitudinal studies in Africa

	Generic WHO TB patient cost survey	Adapted patient cost survey – for TB Sequel
Design	Cross-sectional facility-based	Longitudinal facility-based
Geographic location	National survey	Five African study clinics located in The Gambia, Mozambique, South Africa and Tanzania.
Population	All patients (adults and children) registered for TB (drug-susceptible or drug-resistant) treatment in the National Treatment Programme (NTP).	Adult patients (≥18 years) registered for TB (drug-susceptible or drug-resistant) treatment in the National Treatment Programme (NTP) in the study sites
Sampling	Random cluster sampling or a national simple random sampling where electronic registers are available.	Consecutive sampling – as eligible participants present at the facility [10]
	Sample size based on assumed prevalence of catastrophic costs.	Sample size based on prevalence (>20%) of the outcome of interest which is severe pulmonary function impairment, measured by spirometry at least 2 years after treatment initiation
Enrolment	Consecutive patients registered for TB treatment who are attending a sampled facility and are a minimum of 14 days into either the intensive or continuation phase.	Starting (+/- 14 days) TB treatment after TB diagnosis.
Interview schedule	Each patient is interviewed only once	Patients are interviewed multiple times at defined study visits (M0, M2, M6, M12 and M24).
Resources and time requirements	The survey team is typically comprised of a principal investigator, a survey coordinator, a data manager and interviewers – these are temporary or short-term positions.	The team composition includes data capturers, quality control manager, and a statistician. Permanent or long-term positions are needed for continuity and retention of experienced staff.
	Once off interview and consent (±45 minutes), either paper-based or electronic survey.	Multiple interviews (±45 minutes), only paper-based survey with data entry into an electronic database.
	Qualitative interviews can be performed both with patients and household members, as well as health and social service staff and policy-makers [2].	No qualitative component but includes additional component to study social consequences (e.g. quality of life, pain, disability and stigma related to TB) [10].
Interviewers	Facility-based	Facility- and community-based
Calculating total costs	Requires some retrospective data collection, forward projections and imputations to calculate the total costs.	Total costs available for each phase of TB treatment (i.e., pre-treatment, intensive phase of treatment, continuation phase of treatment and post-TB treatment).
	Only patients in the intensive phase receive questions on the costs incurred prior to diagnosis.	All patients receive questions on the costs incurred prior to diagnosis at the enrolment/M0 visit.
Outcomes	Treatment outcomes are typically not available	Treatment outcomes are available
	Estimate the cost per patient treated for TB	Can estimate the cost per patient treated for TB by outcome Repeated measures of health-related quality of life, pain, depression or anxiety and disability related to TB is collected at M2, M6, M12 and M24. May contribute to better estimates of disability-adjusted life years (DALYs).
Limitations	Survey findings can only be generalized to a subset of people with TB who receive care in the NTP. Conclusions cannot be drawn about all people with TB in the country.	Survey findings can only be generalized to a subset of people with TB who receive care in the NTP in the study sites. Conclusions cannot be drawn about all people with TB in the country.
		The study site may not necessarily be where the patient goes for routine TB care or to collect medication. This may result in issues with recall as participants try to distinguish between routine treatment versus study related visits.
		Direct costs, income loss or coping costs may be underestimated as study sites reimburse patients for travel and time at each visit or may provide support (e.g. food parcels, supplements, travel vouchers) to promote retention.
	Recall bias – a major challenge for the estimation of total patient costs incurred. This mainly affects cost estimates for the pre-treatment period.	To minimize recall bias, patients receive questions on the costs incurred prior to diagnosis at the first visit (M0). During subsequent visits, patients are asked to recall since the date of their last interview. Interviewers are also trained to make it easier for the patient to recall by using local methods of time structuring.
	Costs after treatment completion are not included.	Costs after treatment completion are included. Patients are followed for a minimum of 24 months after TB treatment initiation.
	The current methodology only includes TB patients enrolled and retained in care.	A longitudinal design can only consider earlier aspects of the cascade of care, prior to when a patient becomes lost to follow up.

### Longitudinal adaptation

We first adapted the generic instrument for the longitudinal study design of TB Sequel. We reviewed treatment guidelines for drug-sensitive and drug-resistant TB from the different countries [11–14] and adapted the generic instrument for use at enrollment (Day 0) and at Month 2, Month 6, Month 12 and Month 24, time points intended to capture costs incurred for different phases of TB. These time points make the adapted version suitable for use in patients with either drug-sensitive or drug-resistant TB. It should be noted that these time points were guided by standard treatment durations and do make provision for instances when the treatment duration may be extended (e.g. absence of smear conversion, severe or complicated disease or a treatment interruption lasting less than 2 months) [15].

**Figure 1.**
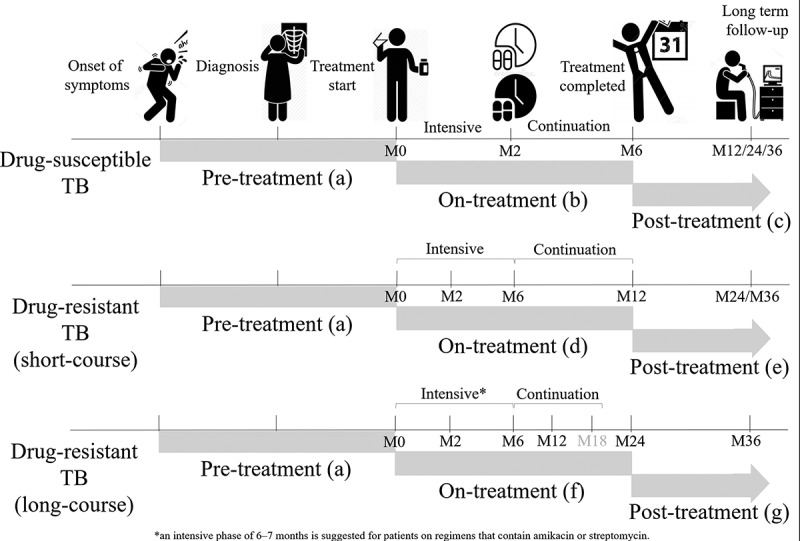
The WHO generic TB patient cost survey instrument was adapted for use at use at enrollment/start of TB treatment (Day 0) and at Month 2, Month 6, Month 12 and Month 24; time points intended to capture costs incurred for different phases of TB disease (e.g. pre-treatment, on-treatment and post-treatment)

Treatment phases for drug-susceptible TB: The preferred regimen for treating adults with TB remains a regimen consisting of an intensive phase of 2 months of isoniazid (INH), rifampin (RIF), pyrazinamide (PZA), and ethambutol (EMB) followed by a continuation phase of 4 months of INH and RIF. Using the time points described above, pre-treatment costs (Figure 1(a)) are estimated by asking patients to recall any out-of-pocket payments or indirect costs that they may have incurred from the onset of TB symptoms to the start of TB treatment (Day 0). Similarly, on-treatment (Figure 1(b)) costs for the intensive and continuation phase of TB treatment are estimated by asking patients to recall any out-of-pocket payments or indirect costs that they may have incurred from the start of TB treatment (Day 0) until Month 2, or from Month 2 to Month 6, respectively. The total on-treatment costs are estimated by combining costs incurred during these two time-points (Month 2 and Month 6). Post-treatment costs are estimated by asking patients to recall any out-of-pocket payments or indirect costs that they may have incurred from the end of TB treatment until the end of long-term follow-up (i.e. Month 12 to capture costs incurred in the first 6 months after treatment completion or Month 24 to capture costs incurred in the year after TB treatment completion) (Figure 1(c)).

Treatment phases for drug-resistant TB: The guidelines for drug-resistant TB were updated by the WHO in 2016 and 2019 to include the use of the shorter multi-drug resistant TB (MDR-TB) regimen (4–6 months intensive phase and 5 months continuation phase) [16,17]. Because the duration of treatment is longer for drug-resistant TB, the Month 2 and Month 6 instruments capture costs for the intensive phase of TB treatment. Then at 12 months, instead of administering the Month 12 instrument to capture post-treatment costs (as we would do for drug-susceptible TB patients), the Month 6 instrument is repeated in drug-resistant TB patients to capture on-treatment costs for the continuation phase. By combining costs incurred during these time-points (Month 2, Month 6 and Month 12), the total on-treatment costs can be estimated (Figure 1(d)). Post-treatment costs incurred in the year after TB treatment completion are collected using the Month 12 instrument (that was used at Month 12 for drug-susceptible TB patients) but administered at Month 24 (Figure 1(e)). The Month 12 instrument (for drug-susceptible TB patients), is administered once patients have completed TB treatment, and can be repeated at multiple time points after TB treatment completion (e.g. 12, 18, 24, 36 months, etc.). The adapted version for these pre-defined time points can be downloaded for use at www.tbsequel.org.

For drug-resistant TB treatment, countries can also opt for an individualized longer (18–20 months or 15–17 months after culture conversion) MDR-TB regimen [17], and preferably an all-oral regimen. In this case, countries can still use the adapted instruments to evaluate and monitor TB’s economic burden on patients and their households. Total on-treatment costs are estimated from costs reported at Month 2, Month 6, Month 12, and Month 24 time-points (Figure 1(f)), however an additional visit is required at Month 18 to minimize recall bias. The Month 6 instrument is repeated until TB treatment has been completed, and then as described above once treatment has been completed, the Month 12 instrument can be administered to collect post-treatment costs. In this instance, it would be important to record the treatment completion date so that the overlap between on-treatment (12–20 months) and post-treatment periods (20–24 months) can be estimated during the analysis phase.

Each time point contains questions for the key data elements described above. While many of the data elements are repeated at the different time points (e.g. employment, numbers of hours worked, use of dissaving strategies, social assistance/protection, guardian costs, etc.), certain sections, specifically the direct out-of-pocket medical and non-medical payments, were adapted for the phase of treatment. For example, the pre- and post-treatment phases do not require data on direct medical (e.g. X-ray, laboratory tests or medication) or non-medical (e.g. transport to collect TB medication) expenditures related to TB treatment and these questions are therefore omitted. Recall data on direct out-of-pocket payments are collected for all the time points of any visit to a health-care provider (whether scheduled or unscheduled, and including outpatient and inpatient care). For on-treatment and post-treatment direct medical and non-medical costs, participants are asked to recall since the date of the last interview. For pre-treatment costs, participants are asked to recall since the date when they first experienced symptoms. Pre-treatment costs (Day 0) are only collected for new TB cases as health-seeking behavior and duration of illness prior to TB treatment, and therefore the pre-treatment costs, are likely to be different for those previously treated (i.e. those with relapse, reinfection or retreatment after treatment failure).

### Local adaptation

Next, we adapted the longitudinal instrument for each setting, to ensure that questions on TB services (e.g. type of provider), health-care fees for TB-specific tests and treatments, health insurance schemes, social protection mechanisms (disability grant, social welfare for the poor, travel vouchers, food assistance), socio-economic status (household assets), and socio-demographic information (education, employment and occupations) are locally appropriate. Discussions were held with local teams to determine the best way to adapt the instruments in terms of language to ensure that words in the target language conveyed the same or similar meaning as the source language (i.e. with an emphasis on thematic translation in local languages rather than literal translation of questions). Adjustments were made by modifying the list of options available for certain questions based on input from local clinicians, counsellors, and researchers. For example, in South Africa and The Gambia, we adapted the list of providers to reflect those commonly used while seeking care (e.g. pharmacy, traditional healer/practitioner, primary care clinic, private practitioner, public hospital, private hospital and herbalist). We also adapted the instrument to include the local currency unit.

As recommended by the WHO Task Force [2], instrument questions on self-reported income and household assets were adapted for each country, where possible using the same wording as in the standardized and validated demographic, expenditure or social survey instruments available for that country (e.g. Demographic or Health Survey or the Household Income and Expenditure Survey).

An interviewer aid with pictograms (e.g. for rating scales), definitions and/or synonyms for certain words or terms and other useful information was developed to supplement the training material and help guide interviewers during the patient interview. A sheet with country-specific prompted income ranges, as described below, was also included in the interviewer aid. The adapted instrument was available in English and was verbally translated into local languages by local study staff, who administer the questionnaire to the study participants. The study team agreed on appropriate wording for the translations during the training and piloting phase (see below), and a summary of this was also included in the interviewer aid. The interviewer aid was also designed to help interviewers with time structuring (e.g. help patients correctly recall costs they incurred since the last interview) and accurate reporting (e.g. help patients accurately recall changes in income since the last interview).

### Income adjustments

Using the longitudinal instrument, participants are interviewed at selected time points to capture costs incurred for different phases of TB disease. Changes in costs and income can then be calculated for the entire TB episode or for each phase of TB (e.g. pre-treatment, on-treatment and post-treatment). The adapted tool allows for multiple methods for income estimation, as recommended by the WHO Task Force, and for alternative measures of income impact, such as the adoption of coping strategies. Measures of annual household income, which is used as the denominator (equivalent to household capacity to pay) when calculating the percentage for which the defined threshold for catastrophic total costs due to TB disease (>20%) is applied [4], can incorporate multiple direct and indirect measures, as described by Sweeney and colleagues and detailed in Table 2 [18].

**Table 2.** Approaches to estimating annual household income using the TB patient cost instrument adapted for longitudinal studies*

Catastrophic total costs^&^ = Episode direct costs^a^ ± Episode indirect cost^b^ > threshold value (e.g. 20% base case) Household capacity to pay^c,d^	
Direct medical and non-medical costs^a^	Total indirect (productivity) loss^b^
These are payments made directly by the patient or their household member. Direct medical payments include payments for formal medical professionals, informal traditional or alternative practitioners, clinics, health centers, pharmacies, and hospitals – for medical services and products (e.g. medicines, consultation fee, payment to DOTS provider/supporter, day charges for hospitalization, diagnostics, lab tests and procedures). This excludes prepayment for health services – for example health insurance premiums – and where relevant, net of any reimbursements to the individual who made the payments. Non-medical direct costs include travel, accommodation, food or other non-medical payments incurred by the patient, their household member or caregiver/guardian while picking up medication or during the visit/hospital stay for TB care (e.g. nutritional or food supplements, interest on loans taken out to meet the costs of TB, day charges for time in hospital etc.).	Indirect costs can be estimated using two alternative methods; Self-reported household income loss net of welfare or social assistance payments. This includes the cost that the caregivers bear by contributing their time and in-kind services. Any gain or loss of income reported during each phase or for the entire episode is considered the total indirect cost [18]. Opportunity cost of time spent away from the daily productive routine. Participants are asked to self-report time spent seeking and receiving care as well as the average number of hours they work each day, if this changed since the last interview, and if so, by how much. The time total period of absence (in hours) is multiplied by the hourly wage rate of the absent worker. The hourly wage can be estimated from directly reported data, household asset ownership, or national statistics.
The sum of the direct medical and non-medical costs for the different phases/entire episode is used as the numerator.	Indirect costs for the different phase/entire episode is used as the numerator in the catastrophic total cost equation.
Household capacity to pay^c,d^	
Self-reported current income (detail or prompted ranges)^c^	Estimated income based on asset scoring^d^
Participants are asked to self-report their monthly individual and household income, which is then used to calculate the annual income. If participants don’t know or refuse to answer they are asked to identify their individual and household income from a list of prompted ranges. Detailed questions are used to solicit information about income (e.g. employment, property income, income from household production of services or goods) and non-salary income (e.g. travel voucher, food vouchers, disability grant, in-kind or cash transfers, public assistance, donations etc.) Annual household income before the onset of symptoms is typically used for the denominator.	Annual household income can be estimated using asset ownership or dwelling characteristics (e.g. number of rooms, type of toilet facility, electricity supply, source of drinking water). A standard asset index can be used to divide households into five socio-economic quintiles. For each income quintile, mean household permanent income can be extrapolated from the National Income Dynamics Survey. This estimate usually represents income before TB. Estimating income based on assets is a suitable alternative if income is hard to report accurately or is subject to great variability over time (e.g. casual or seasonal work, large informal sector work etc.).
Alternative indicator of catastrophic costs – Coping strategies	
Coping strategies can be used as an indicator of economic catastrophe. Participants are asked if the individual or their household used savings, borrowed money, sold property or took out a loan to cover the cover of TB, and if so, how much. Participants are also asked about the economic consequences experienced (e.g. pulling children out of school, household food security etc.)	

*Adapted from [2] and [18].

Abbreviations: TB tuberculosis; DOTS Directly Observed Treatment Short-course & Participants are interviewed at time points intended to capture costs incurred for different phases of TB disease (e.g. pre-treatment, on-treatment and post-treatment). Catastrophic total costs can be calculated for the entire TB episode or for each phase of TB.

a = Direct medical and non-medical costs; b = Total indirect (productivity) loss; c = Self-reported current income (detail or prompted ranges); d = Estimated income based on asset scoring.

To estimate individual and household monthly income, we first asked patients to report their current individual and household monthly income at each time point (e.g. Day 0, Month 2, Month 6, Month 12 and Month 24). At Day 0 patients were also asked to report their individual and household monthly income before the onset of symptoms (6 months ago). At each time point, if patients could not recall their individual or household monthly income or refused to answer, they were asked to identify their individual or household monthly income from country-specific income ranges. Pre-determined weekly, monthly, and annual income bands were included on a sheet, from which study participants could select the most appropriate response. Additional options for ‘none’, ‘don’t know’ and ‘refuse to answer’ were again included to understand missing data. The self-reported mid-point income for the prompted range was used as the current monthly income. The monthly household income reported was then used to calculate the total income for the period (e.g. 2 months, 4 months, or 6 months since the last interview). Similarly, household income was annualized by multiplying the total income for the period by the ratio of the number of months in a year (12) over the number of months in the period. For example, if a patient reported income at two or three time points (e.g. United States Dollar (USD) 2 USD 000 per month at Month 2 and USD 2 USD 200 per month at Month 6), we multiplied the total income for the period (e.g. USD 4 USD 200 for two months) by a ratio (number of months in a year divided by the number of months of income was reported; 12 ÷ 2 = 6) to estimate the amount of income in a year (e.g. USD 25 USD 200 per year). When calculating the proportion of TB patients and their households facing catastrophic total costs, defined as total direct and indirect medical costs exceeding 20% of annual household income, household income prior to symptom onset (6 months prior to treatment initiation) is used as the denominator for catastrophic total costs [18,19].

### Staff training and local refinement

Study staff were trained according to the recommendations outlined in the WHO Task Force and the TB patient cost surveys handbook [2]. Training consisted of an initial two-day introductory session, followed by a second two-day training on interviewing techniques, infection control, ethical considerations, understanding the indicators used in the instrument, and quality assurance (see below). Investigators then conducted an extensive three to five-day training session, either in-country or via interactive virtual training, to ensure that interviewers understood the questions and that the instrument would be implemented in a standardized way. The study team worked with the local study staff to identify potential sources of information (e.g. patient TB card, TB treatment register, etc.), so that clinic record information, such as the date of diagnosis and treatment regimen, could be extracted before the interview.

The study team also worked with local study staff to; (i) list types of health-care providers so that options in the instrument reflected the local typology/categories, (ii) identify country-specific prompted ranges for income, (iii) adapt some of the socio-demographic questions such as education, employment, occupation, so that options reflected the local standard categories, (iv) identify and include types of health insurance and social protection schemes that are available, and (v) adapt questions on income and assets using standardized/validated categories used in the country. Training material and a training guide documenting these adaptions and recommendations were distributed to study staff. Follow-up sessions were scheduled throughout the study period to provide feedback, resolve uncertainties or concerns, and repeat data collection procedures.

We considered the four months between the first introductory training session (May 2017) and the start of enrollment (September 2017) as the pilot phase. During this period, interviewers practiced administering the questionnaires, familiarized themselves with the online data collection tool (OpenClinica®), and provided extensive feedback to the study team, which was then incorporated in the adapted instrument. Countries continued to refine questions to ensure that options were appropriate for their setting (e.g. type of health insurance or social assistance available) and that different context-specific scenarios had been considered in the wording and subsequent options for the questions. For example, for The Gambia, the question on household assets was adapted from ‘Do you own any of the following’ to ‘Does your household/dwelling have or do you own any of the following?’ to reflect local living conditions (e.g. large families living in compounds). Similarly, in instances where patients were not formally employed but traded goods, interviewers were asked to obtain the value of goods, as a proxy for individual monthly income.

## Discussion

The adapted, longitudinal TB cost instrument described above offers several advantages over the original, cross-sectional instrument. Collecting repeated measurements from each subject over time can simultaneously increase statistical power for detecting changes while reducing the costs of conducting a study [20]. Using the adapted tool provides the opportunity to repeat measures and make comparisons over time, describe changes that extend beyond treatment completion, and even link cost survey data to treatment outcomes. These adapted tools are particularly valuable when describing the economic burden of TB during different phases of TB treatment or determining whether economic consequences diminish or reverse when patients recover from TB. The longitudinal adaptation also allows for more in-depth information than does WHO’s generic cross-sectional instrument. For instance, collecting repeated measurements of key variables can provide a more definitive evaluation of within-person change across time [20]. The longitudinal design can be used to further validate the cross-sectional approach and highlight dimensions which are particularly difficult to capture valid data for through extrapolations and imputations. In addition, the adapted tool can be used for either drug-susceptible or drug-resistant TB, making it particularly valuable in view of the scarcity of evidence available on the costs of drug-resistant TB to patients [21].

Additional time and resources, increased risk of attrition, and more complex sample size selection are some of the disadvantages of a longitudinal over a cross-sectional design. While longitudinal studies typically have higher statistical power and require fewer subjects than cross-sectional designs, repeated measurements taken from the same participant are correlated. Therefore, the sample size needs to account for special statistical methods required to analyze correlated data (e.g. multilevel models) [20]. In terms of capturing costs incurred for TB, these two instruments have different purposes. The WHO cross-sectional approach is driven by feasibility and the need to enable as many countries as possible to generate data to monitor the End TB indicator, for which the first milestone year is 2020. Whereas the longitudinal approach was adopted to suit the study design objectives of the research study (e.g. make comparisons over time and capture costs for each phase of treatment spanning from the onset of symptoms until at least 24 months after the completion of treatment). Therefore, it is essential to note that the longitudinal design is preferable for research purposes, while the cross-sectional approach is a more feasible option for national surveillance.

We conclude by noting that both instruments have some limitations (Table 1). It should be noted that the WHO generic TB patient cost survey was adapted for a longitudinal study design and tailored for the TB Sequel study. Therefore, there may be a need to further adapt the instruments for other settings or patient populations (e.g. children or those with extra-pulmonary TB).

The instruments do not capture the impact of mortality associated with TB on the household, nor do they capture the impact of multiple episodes of TB in the same individual or of multiple concurrent illnesses in the same household. Self-reported income can be challenging to assess in settings with informal economies [6,18,22–24]. For example, it is not possible to distinguish lost income due to illness from lost wages while seeking care for patients who report a zero income. Neither instrument measures household consumption or expenditure (i.e. the sum of the monetary values of all items consumed by the household) to estimate annual household income, though questions could be added for settings in which short-form consumption questionnaires have been validated. While the generic and adapted tools can be administered as paper-based surveys with subsequent data entry into an electronic database, the generic instrument has the added advantage that it can be administered as an electronic (E-survey) survey, offering secure management of electronic forms and data in real-time [2]. For those whose research questions are not hampered by these limitations; however, the longitudinal instrument provides a valuable addition to the toolkit for understanding the economic impact of tuberculosis.

## Availability of data and materials

Templates for the adapted instruments (e.g. Day 0, Month 2, Month 6, Month 12 and Month 24) can be freely downloaded from the TB Sequel website (www.tbsequel.org) along with examples of the manual/guide and the interviewer aid which can be adapted. Templates are free to use provided users agree to the following terms and conditions.

TB Sequel hereby grants permission to use the adapted patient cost instruments under the following conditions, which shall be assumed by all to have been agreed to as a consequence of accepting and using the documents:

- Changes can be made to the instruments; however, all such changes shall be identified as having been made by the user.
- The user accepts full responsibility, and agrees to indemnify and hold TB Sequel harmless, for the accuracy of any translations into another language and any errors, omissions, misinterpretations, or consequences thereof, and any consequences resulting from the use of the instrument.
- The user will provide a credit line when printing and distributing the instruments acknowledging that it was developed as part of the TB Sequel study and acknowledging this and the sources; the WHO’s generic TB patient cost instrument and the TB patient cost surveys handbook.

## Paper context

The WHO cross-sectional TB patient cost tool was developed to enable countries to monitor the End TB indicator, namely, to eliminate the number of TB-affected households facing catastrophic costs by 2035. We adapted the generic tool, typically designed for national surveillance studies, for longitudinal studies. The adapted tool can now be used to make comparisons over time, describe changes that extend beyond treatment completion, and link cost survey data to treatment outcomes and post-TB sequelae.

## ORCID details

Van Rensburg (craigvr84@gmail.com; ORCID identifier 0000-0003-0458-3306),

Govathson (cgovathson@heroza.org; ORCID identifier 0000-0003-2595-1913),

Ivanova (Olena.Ivanova@lrz.uni-muenchen.de; ORCID identifier 0000-0002-8276-7891),

Rieß (Friedrich.Riess@lrz.uni-muenchen.de),

Siroka (sirokaa@who.int; ORCID identifier 0000-0002-0320-0775),

Sillah K (asillah@mrc.gm; ORCID identifier 0000-0001-8332-2475),

Ntinginya (nelias@nimr-mmrc.org; ORCID identifier 0000-0002-1575-4521),

Jani (ilesh.jani@gmail.com; ORCID identifier 0000-0002-6880-6655),

Sathar (FSathar@auruminstitute.org; ORCID identifier 0000-0002-8296-8554),

Rosen (srosen@bu.edu; ORCID identifier 0000-0002-6560-2964),

Sanne (isanne@witshealth.co.za; ORCID identifier 0000-0003-4729-3405),

Rachow (rachow@lrz.uni-muenchen.de; ORCID identifier 0000-0002-4269-3699),

Lönnroth (knut.lonnroth@ki.se; ORCID identifier 0000-0001-5054-8240).

## TB Sequel Consortium members

Gavin Churchyard, Michael Hoelscher, Robert Wallis, Salome Charalambous, Kavindhran Velen, Farzana Sathar, Fadzai Munedzimwe, Andrea Rachow, Olena Ivanova, Anna-Maria Mekota, Abhishek Bakuli, Christof Geldmacher, Friedrich Riess, Fidelina Zekoll, Ulrich von Both, Knut Lönnroth, Stefan Nimann, Matthias Merker, Viola Dreyer, Ulrich Schaible, Christoph Leschczyk, Beate Kampmann, Jayne Sutherland, Basil Sambou, Caleb Muefong, Fatoumatta Darboe, Abi-Janet Riley, Binta Sarr, Olumuyiwa Owolabi, Ben Dowsing, Azeezat Sallahdeen, Shamanthi Jayasooriya, Abdou K Sillah, Nyanda Elias Ntinginya, Issa Sabi, Daniel Mapamba, Mkunde Chachage, Elimina Siyame, Julieth M. Lalashowi, Ian Sanne, Mohammed Rassool, Lyndel Singh, Jaclyn Bennet, Noluthando Mwelase, Ilesh Jani, Celso Khosa, Nilesh Bhatt, Sofia Viegas, Khalide Azam, Nadia Sitoe, Pedroso Nhassengo, Denise Evans, Kamban Hirasen, Caroline Govathson, Tembeka Sineke, Sydney Rosen.
